# Agreement and potential for arithmetic adjustment of anterior segment measurements across IOLMaster 700, Pentacam HR, and Sirius

**DOI:** 10.1038/s41598-026-42204-9

**Published:** 2026-03-26

**Authors:** Armin Doostparast, Farbod Semnani, Maryam Ghandhari, Mohammadreza Ghandhari, Amir Hossein Khosronejad, Ali Ahmadi, Alireza Eslampoor

**Affiliations:** 1 Eye Research Center, Mashhad University of Medical Sciences, Mashhad, Iran; 2 Student Research Committee, Faculty of Medicine, Mashhad University of Medical Sciences, Mashhad, Iran; 3 School of Medicine, Tehran University of Medical Sciences, Tehran, Iran; 4 National Center for Health Insurance Research, Tehran, Iran; 5 Department of Radiology, School of Medicine, Mashhad University of Medical Sciences, Mashhad, Iran; 6 Student Research Committee, Mazandaran University of Medical Sciences, Sari, Iran

**Keywords:** IOLMaster 700, Pentacam HR, Sirius, WTW, Agreement, ICL sizing, Phakic IOL, Diseases, Health care, Medical research

## Abstract

We aimed to evaluate the absolute agreement, consistency, and the potential of arithmetic adjustment for measurements across a swept-source optical coherence tomography biometer (IOLMaster 700), a single Scheimpflug-based tomographer (Pentacam HR), and a combined Scheimpflug-Placido disc-based tomographer (Sirius). This cross-sectional study analyzed the corneal keratometry parameters (Sim-K1, Sim-K2, and Sim-Km, corneal astigmatism (CA), and its Jackson power vectors (J0 and J45), central corneal thickness (CCT), anterior chamber depth (ACD), and the horizontal white-to-white distance (WTW) of 111 healthy eyes. The inter-device agreement was assessed using the Bland-Altman limits of agreement (LoA) and two types of intraclass correlation coefficient (ICC) analyses. A mean difference (MD) translating to less than 0.25D in refractive outcomes was considered clinically acceptable. Except for the WTW (ICC(2,1) = 0.52, ICC(3,1) = 0.65), the inter-device agreement was excellent for all parameters (ICC(2,1) and ICC(3,1) > 0.90), with the CA and its power vectors (ICC(2,1) and ICC(3,1): 0.87–0.90) being slightly inferior. WTW values varied substantially across devices, but applying a constant adjustment (MD = 0.36) between Pentacam HR and Sirius improved agreement (ICC(2,1) rising from 0.59 to 0.90), whereas adjustment was not feasible with IOLMaster 700. CCT and ACD may be considered interchangeable across the three, particularly in intraocular lens (IOL) power calculation (the widest LoA were − 0.20 to 0.16 mm for ACD and − 10.99 to 25.42 μm for CCT, respectively). Keratometry values exhibited clinically relevant LoA (the widest LoA for Sim-Km: − 0.77 to 0.80 diopters), limiting their direct interchangeability. Although WTW showed the greatest variability (the widest LoA: − 0.86 to 0.64 mm), the agreement between Pentacam HR and Sirius could be improved through an arithmetic adjustment. This adjustment indicated a systematic error rather than random variability between Pentacam HR and Sirius. These findings highlight the potential of arithmetic adjustment of WTW, an important variable in implantable collamer lens sizing, similar to constant optimization in the IOL power formulas.

## Introduction

Achieving desirable outcomes in cataract and refractive surgeries heavily depends on the precise measurement of corneal and anterior segment parameters, as minor disparities in these measurements can heavily influence surgical outcomes and the effectiveness of lens implantations^1^. In this sense, various imaging technologies have been developed to improve the accuracy of these measurements, including corneal topography, ocular biometry, or more recently, optical coherence tomography (OCT). Imaging systems even combine various technologies to enhance the diagnostic accuracy.

The Scheimpflug camera is one of the most accurate and widely used technologies, which was developed to precisely measure the corneal parameters. Pentacam (OCULUS Optikgeräte GmbH, Wetzlar, Germany) is one of the most commonly used devices in clinics that uses a single rotating Scheimpflug camera to capture detailed images from different angles while a monochromatic 475 nm slit-light source illuminates the cornea. These data are further analyzed to construct a three-dimensional model from up to 138,000 elevation points of the cornea, allowing the clinicians to make a fully precise assessment of the anterior segment^2,3^. Sirius (CSO, Florence, Italy) employs a combination of a single Scheimpflug camera and a Placido disc-based topographer of 22 rings, along with a 475-nm blue LED light. The Scheimpflug system can capture 25 radial sections of the cornea with one Placido top-view image in approximately a 5–6-s acquisition period, covering a 12 mm area of the cornea. The anterior corneal surface data from both the Placido and Scheimpflug images are merged using a proprietary method. Additionally, all measurements for the internal structures are derived exclusively from the Scheimpflug data^4–8^. The IOLMaster 700 (Carl Zeiss, Jena, Germany) is a non-invasive optical biometer that uses swept Source Optical Coherence Tomography (SS-OCT) to produce two-dimensional OCT cross-sectional eye scans. It operates at wavelengths ranging from 1035 to 1080 nm, with a scanning rate of 2 kHz and a scan depth of 44 mm. The images are generated based on the longitudinal information from the cornea to the retina in six meridians: 0°, 30°, 60°, 90°, 120°, and 150°. Each meridional scan is created by averaging three individual scans, and it is used to obtain all essential axial biometry measurements^9,10^.

The keratometry parameters, along with pachymetry and biometric indices, including ACD anterior chamber depth (ACD), central corneal thickness (CCT), and horizontal white-to-white distance of cornea (WTW), are among the most widely used anterior segment parameters in intraocular lens (IOL) power calculation formulas, refractive, and implantable collamer lens (ICL) surgeries planning^11–13^.

It is necessary that the agreement of measurement obtained from different systems be assessed to ensure the interpretation of their data is valid and whether their measurements could be used interchangeably, particularly across visits. Pentacam HR, Sirius, and IOL master 700 imaging devices were investigated in the current study due to their widespread use in clinics. Although the agreement of measurements derived from these devices has already been widely investigated, to the best of our knowledge, the current literature on agreement studies largely lacks a universal methodology, the distinction between clinical and statistical significance is sometimes unclear, and the potential for arithmetic adjustment in cases of poor agreement has been rarely explored. Hence, we aimed to conduct a comprehensive agreement analysis to shed light on the different aspects of the measurements’ agreement between these devices and address their clinical interchangeability.

### Study design and ethics

This cross-sectional study was conducted at the Noorafarin Eye Clinic from December 2023 to February 2024. The institutional review board of Mashhad University of Medical Sciences approved the study protocol, and the permission was registered under the following number: IR.MUMS.MEDICAL.REC.1403.238. All procedures in the study were performed in accordance with the principles of the Declaration of Helsinki of 1975 (revised in 1983). After fully explaining the study procedures and goals, written informed consent was gathered from all of the participants.

## Methods

This study involved 111 left virgin eyes of 111 individuals who were candidates for keratorefractive surgery. To closely align patients based on their demographic profiles, all participants were selected from the same ethnicity.

The exclusion criteria included the following: poor fixation, any corneal pathology (e.g., a history of confirmed or suspected keratoconus), previous ocular surgery, corneal scarring, glaucoma, confirmed macular pathology, severe ocular trauma, use of rigid contact lenses within the past month, or use of soft contact lenses within the week preceding the examination.

All of the participants underwent a full optometric examination, followed by a complete set of anterior segment imaging using the Pentacam HR (software version 1.21r.65), Sirius (phoenix v3.7.01.08), and ZEISS IOLMaster 700 (software version 1.50) devices.

The imaging protocol for each device was as follows: The participants were asked to put their chin on the chin rest and slightly push their forehead against the forehead strap. Subsequently, they were asked to blink fully to establish a proper tear film on the cornea. They were also instructed not to move their head or blink while the device was operating, staring directly at the center of the light source while keeping their eyes wide open. Meanwhile, a cover was placed on their head to provide a fully dark environment, which was only disturbed by the light beams emitted during the device’s operation to ensure proper and equal lighting conditions for measuring the pupil diameter. All measurements were device-automated only after the operator had manually positioned the screen marker of each device at the center of the cornea and initiated the automatic operation. The patients underwent ocular imaging in the following sequence: Pentacam HR, Sirius, and then IOLMaster 700. A high-quality acquisition, verified by the internal quality check assessment, was retrieved from each instrument for analysis. The acquisition was repeated if the in-system scanning quality was not deemed appropriate. The same professional operator performed the imaging protocol, with at least five-minute intervals between acquisitions to prevent visual asthenopia or tear-film instability. All participants were examined between 10 am and 12 pm. The operator performed daily calibrations according to the manufacturer’s instructions to ensure the measurement’s precision and reliability.

Keratometry parameters, WTW, CCT, and ACD were recorded for each patient. Keratometry measurements consisted of simulated flat keratometry (Sim-K1), simulated steep keratometry (Sim-K2), simulated mean keratometry (Sim-Km), and corneal astigmatism (CA). Moreover, power vector analysis was conducted for the CA to obtain the regular component at 0°/90° and the oblique component at 45°/135°, known as J0 and J45, respectively. The Sim-K1 and Sim-K2 were calculated solely based on the radii of the anterior curvature of the cornea and the customary refractive index of 1.3375. To calculate the simulated values, the Pentacam HR and Sirius measure the radii of the anterior surface of the cornea in a 3 mm circular zone centered at the corneal vertex, while the IOLMaster 700 covers an approximately 2.5 mm circular zone^14,15^. The other keratometry parameters were derived based on the following formulas (θ stands for the astigmatic axis):

$${\text{Sim - Km}} = \frac{{{\text{Sim - K1}} + {\text{Sim - K2}}}}{{\mathrm{2}}}$$

$${\mathrm{CA}} = {\text{Sim - K1}} - {\text{Sim - K2}}$$

$${\mathrm{J0}} = \frac{{ - {\mathrm{1*CA}}}}{{\mathrm{2}}} \cdot {\mathrm{cos}}\left( {{\mathrm{2}}\theta } \right)$$

$${\mathrm{J45}} = \frac{{ - {\mathrm{1*CA}}}}{{\mathrm{2}}} \cdot {\mathrm{sin}}\left( {{\mathrm{2}}\theta } \right)$$

The ACD was defined as the distance measured from the corneal epithelium to the anterior surface of the crystalline lens^16^. It is worth noting that Sirius employs a different convention to display some parameters, as WTW is marked as HVID (Horizontal Visible Iris Diameter), and ACD is not shown directly but as the arithmetic sum of the aqueous depth and CCT (AD + CCT). Moreover, Pentacam HR reports two different ACD values, ACD(int) and ACD(ext), which align with our definition of aqueous depth and ACD, respectively.

### Statistical analysis

Data analysis and visualization were carried out in Python 3.11, utilizing the following libraries: Pandas (version 2.3.2), Numpy (version 1.24.0), Matplotlib (version 3.7.2), Pingouin (version 0.5.4), and Seaborn (version 0.12.2).

The required sample size (n) was calculated according to the following formula (s = standard deviation of difference):

$$desired\;confidence\;interval\;of\;Limits\;of\;agreement\left( {LoA} \right) = 1.96*\sqrt {\frac{{3s^{2} }}{n}}$$

Using the aforementioned formula, a study by McAlinden et al. recommended that a sample size of 100 subjects would yield a 95% confidence interval (95% CI) for the LoA of 0.34s, which could generally be considered accurate. Therefore, they suggested that agreement studies should include at least 100 subjects to obtain reliable findings^17^. Our study did not have a single endpoint; instead, it involved various parameters with different distributions across the three instruments. As a result, the minimum sample size in the current study was determined to be at least 100.

We compared the means of the measurements across the three systems using the repeated-measures analysis of variances (ANOVA) and pairwise paired-samples t-tests, followed by calculating the effect size (Hedges’ g) to shed light on the magnitude of the difference^18^. Thresholds of < 0.2, 0.2 to 0.5, 0.5 to 0.8, and > 0.8 were chosen to represent negligible, small, medium, and large magnitudes of effect, respectively^19^. A *P* value less than 0.05 was considered statistically significant.

The agreement analysis across the devices was performed using two types of intraclass correlation coefficient (ICC), according to the convention of McGraw and Wong^20^. ICC(2,1) (agreement), which is based on the assumption of two-way random effects, absolute agreement, and single rater/measurement, considers both systematic and random measurement errors and evaluates whether the measurements from one set al.ign with those from the other set. On the other hand, ICC(3,1) (consistency) is based on a two-way mixed effects, consistency, single rater/measurement assumption and examines the consistency of measurements, emphasizing the correlation between the measurements rather than their agreement. First, an overall ICC (ICC(2,1) or ICC(3,1)) was calculated across the three devices to provide an overall estimate of inter-device reliability by partitioning the total variance into between-subject and measurement components (Table 1). Then, the pairwise comparisons were made to shed light on the details of inter-device agreement (Table 2).

**Table 1:** Overall inter-device agreement of measurements across IOLMaster 700, Pentacam HR, and Sirius devices.

Measurement^a^	ICC(2,1)^b^	ICC(3,1)^c^
WTW	0.52 (0.24–0.69)	0.65 (0.56–0.73)
ACD	0.97 (0.96–0.98)	0.98 (0.97–0.98)
CCT	0.95 (0.90–0.97)	0.96 (0.95–0.97)
Sim-K1	0.95 (0.92–0.96)	0.95 (0.93–0.96)
Sim-K2	0.97 (0.96–0.98)	0.98 (0.97–0.98)
Sim-Km	0.97 (0.95–0.98)	0.97 (0.96–0.98)
CA	0.90 (0.86–0.92)	0.90 (0.86–0.92)
CA (J0)	0.87 (0.82–0.90)	0.87 (0.82–0.90)
CA (J45)	0.88 (0.83–0.91)	0.88 (0.84–0.91)

^a^Each measurement; ACD: Anterior Chamber Depth, WTW: White-to-White diameter of cornea, CCT: Central Corneal Thickness, Sim-K1: Flat Keratometry, Sim-K2: Steep Keratometry, Sim-Km: Mean Keratometry, CA: Corneal Astigmatism, CA (J0): Regular component of Corneal Astigmatism (0°/90°), CA (45): Oblique component of Corneal Astigmatism (45°/135°).

^b^ICC(2,1): Intraclass correlation coefficient (2,1) shows the agreement of measurements across the three systems. The numbers within the parentheses present the 95% confidence Intervals.

^c^ICC(3,1): Intraclass correlation coefficient (3,1) shows the consistency of measurements across the three systems. The numbers within the parentheses present the 95% confidence Intervals.

*The *P* values for all ICC values were < 0.001.

**Table 2:** Pairwise inter-device agreement of measurements across IOLMaster 700, Pentacam HR, and Sirius devices.

Measurement^a^	Devices (A-B)^b^	ICC(2,1)^c^	ICC(3,1)^d^	Lower LoA^e^	Upper LoA^e^	LoA width^e^
WTW	P-S	0.59 (-0.07 : 0.86)	0.90 (0.86 : 0.93)	− 0.66 (− 0.71 : − 0.61)	− 0.06 (− 0.11 : − 0.01)	0.60
	I-S	0.48 (0.31 : 0.61)	0.49 (0.34 : 0.62)	− 0.86 (− 0.99 : − 0.74)	0.64 (0.51 : 0.76)	1.50
	I-P	0.47 (0.13 : 0.68)	0.58 (0.44 : 0.69)	− 0.41 (− 0.52 : − 0.30)	0.91 (0.80 : 1.02)	1.32
ACD	P-S	0.97 (0.95 : 0.98)	0.97 (0.95 : 0.98)	− 0.20 (− 0.23 : − 0.17)	0.16 (0.13 : 0.19)	0.36
	I-S	0.97 (0.91 : 0.99)	0.98 (0.97 : 0.98)	− 0.20 (− 0.22 : − 0.17)	0.10 (0.08 : 0.13)	0.30
	I-P	0.98 (0.97 : 0.99)	0.98 (0.97 : 0.99)	− 0.16 (− 0.18 : − 0.14)	0.10 (0.08 : 0.13)	0.26
CCT	P-S	0.95 (0.91 : 0.97)	0.96 (0.94 : 0.97)	− 12.87 (− 15.64 : − 10.10)	20.45 (17.68 : 23.22)	33.32
	I-S	0.93 (0.74 : 0.97)	0.95 (0.93 : 0.97)	− 10.99 (− 14.02 : − 7.97)	25.42 (22.39 : 28.45)	36.41
	I-P	0.96 (0.93 : 0.98)	0.97 (0.96 : 0.98)	− 10.41 (− 12.71 : − 8.11)	17.26 (14.96 : 19.56)	27.67
Sim-K1	P-S	0.95 (0.91 : 0.97)	0.95 (0.93 : 0.97)	− 1.10 (− 1.25 : − 0.95)	0.73 (0.58 : 0.89)	1.83
	I-S	0.93 (0.91 : 0.95)	0.93 (0.91 : 0.95)	− 1.11 (− 1.30 : − 0.93)	1.12 (0.93 : 1.30)	2.23
	I-P	0.96 (0.92 : 0.97)	0.96 (0.95 : 0.97)	− 0.64 (− 0.77 : − 0.50)	1.01 (0.87 : 1.15)	1.65
Sim-K2	P-S	0.98 (0.95 : 0.99)	0.98 (0.97 : 0.99)	− 0.76 (− 0.86 : − 0.66)	0.43 (0.33 : 0.53)	1.19
	I-S	0.97 (0.96 : 0.98)	0.97 (0.96 : 0.98)	− 0.67 (− 0.79 : − 0.56)	0.73 (0.61 : 0.85)	1.40
	I-P	0.97 (0.93 : 0.99)	0.98 (0.97 : 0.99)	− 0.40 (− 0.50 : − 0.30)	0.78 (0.69 : 0.89)	1.18
Sim-Km	P-S	0.97 (0.92 : 0.98)	0.98 (0.97 : 0.98)	− 0.80 (− 0.91 : − 0.70)	0.43 (0.33 : 0.53)	1.23
	I-S	0.96 (0.95 : 0.97)	0.96 (0.95 : 0.97)	− 0.77 (− 0.90 : − 0.64)	0.80 (0.67 : 0.93)	1.57
	I-P	0.97 (0.90 : 0.99)	0.98 (0.98 : 0.99)	− 0.32 (− 0.40 : − 0.23)	0.72 (0.64 : 0.81)	1.04
CA	P-S	0.90 (0.85 : 0.93)	0.90 (0.85 : 0.93)	− 0.98 (− 1.14 : − 0.83)	0.90 (0.74 : 1.06)	1.88
	I-S	0.89 (0.84 : 0.92)	0.89 (0.84 : 0.92)	− 1.04 (− 1.21 : − 0.87)	0.98 (0.81 : 1.14)	2.02
	I-P	0.91 (0.87 : 0.94)	0.91 (0.87 : 0.93)	− 0.91 (− 1.07 : 0.76)	0.93 (0.78 : 1.08)	1.85
CA (J0)	P-S	0.84 (0.78 : 0.89)	0.84 (0.78 : 0.89)	− 0.65 (− 0.77 : − 0.54)	0.73 (0.61 : 0.84)	1.38
	I-S	0.86 (0.80 : 0.90)	0.86 (0.80 : 0.90)	− 0.63 (− 0.74 : − 0.52)	0.68 (0.58 : 0.79)	1.31
	I-P	0.91 (0.87 : 0.93)	0.91 (0.87 : 0.93)	− 0.54 (− 0.63 : − 0.45)	0.52 (0.43 : 0.61)	1.06
CA (45)	P-S	0.88 (0.83 : 0³.92)	0.88 (0.83 : 0.92)	− 0.36 (− 0.41 : − 0.30)	0.32 (0.27 : 0.38)	0.68
	I-S	0.87 (0.81 : 0.91)	0.87 (0.82 : 0.91)	− 0.33 (− 0.39 : − 0.27)	0.41 (0.35 : 0.47)	0.74
	I-P	0.89 (0.82 : 0.92)	0.90 (0.85 : 0.93)	− 0.27 (− 0.33 : − 0.22)	0.39 (0.34 : 0.45)	0.67

^a^Each measurement; ACD: Anterior Chamber Depth, WTW: White-to-White diameter of cornea, CCT: Central Corneal Thickness, Sim-K1: Simulated Flat Keratometry, Sim-K2: Simulated Steep Keratometry, Sim-Km: Simulated mean Keratometry, CA: Corneal Astigmatism, CA (J0): Regular component of Corneal Astigmatism (0°/90°), CA (45): Oblique component of Corneal Astigmatism (45°/135°); all keratometry values are reported as diopter, ACD, and WTW values are reported as mm, and CTT values are reported as micrometer.

^b^P: Pentacam HR, S: Sirius, I: IOLMaster 700.

^c^ICC(2,1): Intraclass correlation coefficient (2,1) shows the agreement of measurements across the three systems. The numbers within the parentheses present the 95% confidence Intervals.

^d^ICC(3,1): Intraclass correlation coefficient (3,1) shows the consistency of measurements across the three systems. The numbers within the parentheses present the 95% confidence Intervals.

^e^Upper and lower LoA: upper and lower Limits of Agreement. The numbers within the parentheses present the 95% confidence Intervals. The LoA width was calculated as follows: upper LoA – lower LoA.

*The *P* values for all ICC values were < 0.001.

The interpretation of ICC values is contextual, yet this study categorized ICC values of < 0.50, 0.50 to 0.74, 0.75 to 0.89, and 0.90 to 1 as representative of poor, moderate, good, and excellent agreement, respectively^21^. If two sets of measurements show disparity in their values through a constant mean difference (MD), but the ranking of observations is preserved, ICC(3,1) remains high^20–22^. A large discrepancy between these two types of ICCs indicates a fixed systematic bias and suggests the potential for adjustment^23,24^. In this case, we first checked for potential proportional bias (varying bias considering different ranges of the variable of interest). It was done by fitting a simple regression line to the scatter plot of pairwise measurement differences and checking whether its slope (beta coefficient) differs significantly from zero. If not (proportional bias precluded), a constant (equal to MD) was added to each measurement of the device with the lower mean measurement^25^.

The Bland-Altman analysis, along with the calculation of the LoA and their corresponding 95% CI, were the final steps in the agreement analysis. Bland-Altman analysis is the most reliable method for evaluating the clinical interchangeability of measurements and was therefore employed to assess the interchangeability of findings before and after potential adjustments.

## Results

This study included 111 healthy left eyes of 111 participants, with an average age of 41.4 ± 5.1 years, consisting of 34 men (31%) and 77 women (69%). The mean subjective spherical refraction was − 1.01 ± 2.38 D (diopters) (range: − 5.98 to 4.45), the mean ocular astigmatism was − 1.31 ± 1.13 D (range: − 4.45 to − 0.13), and the mean axial length was 23.85 ± 1.18 mm (range: 21.18 to 26.53), respectively. The logMAR (Logarithm of the Minimum Angle of Resolution) visual acuity was also 0.01 ± 0.03.

According to Table 1, which demonstrates the overall agreement and consistency of measurements across the three systems, except for the WTW, agreement was excellent for all parameters (ICC(2,1) and ICC(3,1) > 0.90). Notably, the agreement of CA and its power vectors remained good, though slightly inferior compared to the other parameters (ICC(2,1) and ICC(3,1): 0.87–0.90). On the other hand, the agreement (ICC(2,1) = 0.52, 95% CI = [0.24–0.69]) and consistency of measurements (ICC(3,1) = 0.65, 95% CI = [0.56–0.73]) were revealed to be considerably poorer for the WTW. This indicated a relatively moderate inter-device agreement, with no notable improvement in the ICC(3,1) compared to the ICC(2,1).

Table 2 provides more details on the pairwise agreement analysis across devices. Except for WTW, the pairwise agreement between instruments for other measurements was relatively similar to their corresponding overall agreement. For WTW, the ICC(2,1) values were generally close across all pairwise comparisons, ranging from 0.47 to 0.59, indicating relatively moderate agreement. However, the ICC(3,1) values differed significantly across the comparisons, ranging from a still moderate correlation of 0.49 for IOLMaster 700-Sirius and 0.58 for IOLMaster 700-Pentacam HR to an excellent correlation of 0.90 for Pentacam HR-Sirius. This large gap between the ICC values of Pentacam HR-Sirius and IOLMaster 700-Sirius, compared with IOLMaster 700-Pentacam HR, suggests a fixed systematic bias between their WTW measurements. After ruling out the presence of proportional bias (beta-coefficient = − 0.08, *P* value > 0.05) and adding a constant of 0.36 mm to each Pentacam HR WTW measurement, lCC(2,1) rose from 0.59 to 0.90.

**Fig. 1:**
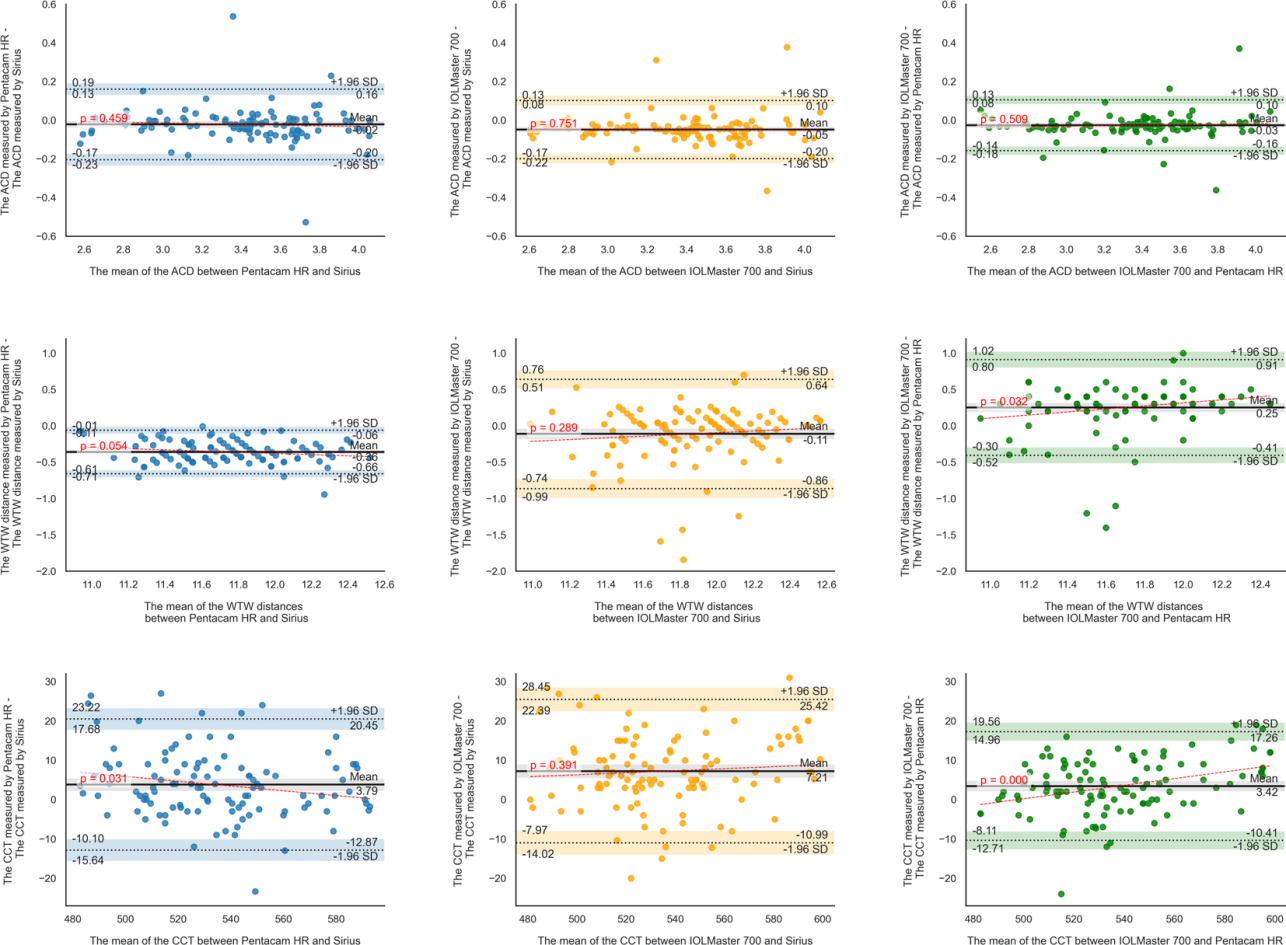
Bland-Altman plots illustrating absolute inter-device agreement (clinical interchangeability) of anterior chamber depth (ACD), horizontal corneal white-to-white distance (WTW), and central corneal thickness (CCT). The red dashed line represents the linear regression of the differences across the measurement range, indicating trends and potential proportional bias.

**Fig. 2:**
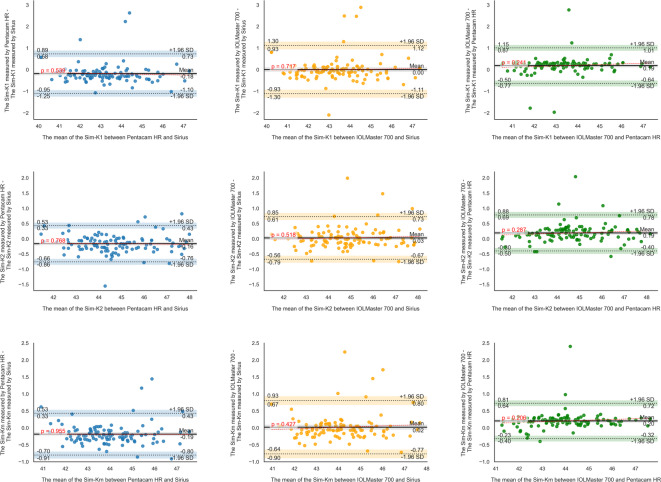
Bland-Altman plots illustrating absolute inter-device agreement (clinical interchangeability) of simulated flat keratometry (Sim-K1), simulated steep keratometry (Sim-K2), and simulated mean keratometry (Sim-Km). The red dashed line represents the linear regression of the differences across the measurement range, indicating trends and potential proportional bias.

**Fig. 3:**
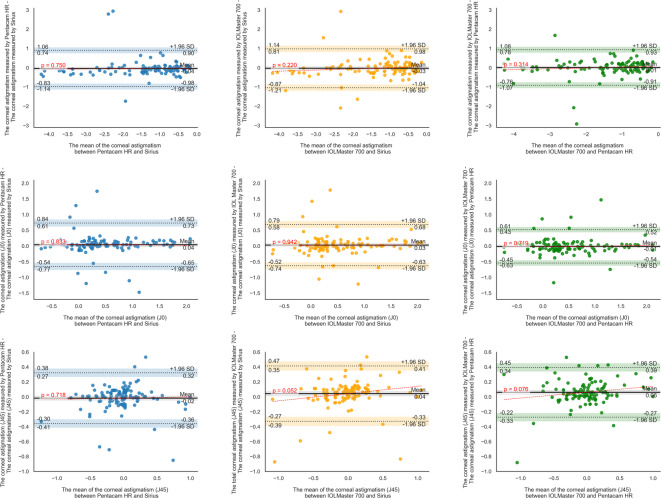
Bland-Altman plots illustrating absolute inter-device agreement (clinical interchangeability) of corneal astigmatism (CA) and the corresponding Jackson power vectors (CA (J0) and CA (J45)). The red dashed line represents the linear regression of the differences across the measurement range, indicating trends and potential proportional bias.

Moreover, the Bland-Altman analysis revealed that for Pentacam HR-Sirius, the LoA ranged from − 12.9 to 20.5 μm for CCT, − 0.2 to 0.16 mm for ACD, − 0.66 to − 0.06 mm for WTW, − 1.1 to 0.7 D for Sim-K1, − 0.8 to 0.4 D for Sim-K2 and Sim-Km, − 1.0 to 0.9 D for CA, − 0.7 to 0.7 D for CA (J0), and − 0.4 to 0.3 D for CA (J45). For IOLMaster 700-Sirius, the LoA ranged from − 11.0 to 25.4 μm for CCT, − 0.2 to 0.1 mm for ACD, − 0.86 to 0.64 mm for WTW, − 1.1 to 1.1 D for Sim-K1, − 0.7 to 0.7 D for Sim-K2, − 0.8 to 0.8 D for Sim-Km, − 1.0 to 1.0 D for CA, − 0.6 to 0.7 D for CA (J0), and − 0.3 to 0.4 D for CA (J45). For IOLMaster 700-Pentacam HR, the LoA ranged from − 10.4 to 17.3 μm for CCT, − 0.16 to 0.1 mm for ACD, − 0.41 to 0.91 mm for WTW, − 0.6 to 1.0 D for Sim-K1, − 0.4 to 0.8 D for Sim-K2, − 0.3 to 0.7 D for Sim-Km, − 0.9 to 0.9 D for CA, − 0.5 to 0.5 D for CA (J0), and − 0.3 to 0.4 D for CA (J45) (Table 2; Figs. 1, 2 and 3).

Table 3 presents a detailed device-by-device pairwise comparison for all measurements. A minor disparity was noted in the keratometry and CA parameters among the devices, with the difference being minimal and negligible (Hedges’ g < 0.2). Moreover, slightly more pronounced differences were observed in the CCT and ACD measurements between the IOLMaster 700 and Sirius (Hedges’ g = 0.24 and − 0.14, respectively) compared to Pentacam HR-Sirius and IOLMaster 700-Pentacam HR comparisons. However, a substantial statistically significant disparity was identified in the WTW measurements between the Pentacam HR and Sirius (11.57 ± 0.34 vs. 11.93 ± 0.36; *P* < 0.001, Hedges’ g = − 1.03), previously highlighted as potentially adjustable. Additionally, moderate to large disparities were observed in the WTW measurements between the IOLMaster 700 and the other two devices; however, no practical arithmetic adjustments could be proposed due to the ICC(3,1) values (Table 2).

**Table 3:** The pairwise comparison of measurements across IOLMaster 700, Pentacam HR, and Sirius devices.

Measurement ^a^	Devices (A-B)^b^	A mean ± SD^c^	B mean ± SD^c^	MD ± SE^d^	*P*-value^e^	Hedges^f^
WTW	P-S	11.57 ± 0.34	11.93 ± 0.36	− 0.36 ± 0.01	< 0.001*	− 1.03
	I-S	11.82 ± 0.40	11.93 ± 0.36	− 0.11 ± 0.04	< 0.01*	− 0.29
	I-P	11.82 ± 0.40	11.57 ± 0.34	0.25 ± 0.03	< 0.001*	0.68
ACD	P-S	3.39 ± 0.36	3.41 ± 0.36	− 0.02 ± 0.01	0.04*	− 0.06
	I-S	3.36 ± 0.36	3.41 ± 0.36	− 0.05 ± 0.01	< 0.001*	− 0.14
	I-P	3.36 ± 0.36	3.39 ± 0.36	− 0.03 ± 0.01	< 0.001*	− 0.07
CCT	P-S	536.04 ± 28.07	532.25 ± 29.79	3.79 ± 0.81	< 0.001*	0.13
	I-S	539.47 ± 30.55	532.25 ± 29.79	7.21 ± 0.88	< 0.001*	0.24
	I-P	539.47 ± 30.55	536.04 ± 28.07	3.42 ± 0.67	< 0.001*	0.12
Sim-K1	P-S	43.23 ± 1.53	43.41 ± 1.55	− 0.18 ± 0.04	< 0.001*	− 0.12
	I-S	43.41 ± 1.57	43.41 ± 1.55	0.00 ± 0.05	1	0.00
	I-P	43.41 ± 1.57	43.23 ± 1.53	0.19 ± 0.04	< 0.001*	0.12
Sim-K2	P-S	44.64 ± 1.53	44.80 ± 1.54	− 0.16 ± 0.03	< 0.001*	− 0.11
	I-S	44.83 ± 1.56	44.80 ± 1.54	0.03 ± 0.03	1	0.02
	I-P	44.83 ± 1.56	44.64 ± 1.53	0.19 ± 0.03	< 0.001*	0.12
Sim-Km	P-S	43.92 ± 1.45	44.11 ± 1.45	− 0.19 ± 0.03	< 0.001*	− 0.13
	I-S	44.12 ± 1.48	44.11 ± 1.45	0.02 ± 0.04	1	0.01
	I-P	44.12 ± 1.48	43.92 ± 1.45	0.20 ± 0.03	< 0.001*	0.14
CA	P-S	− 1.43 ± 1.06	− 1.39 ± 1.05	− 0.04 ± 0.05	1	− 0.04
	I-S	− 1.42 ± 1.11	− 1.39 ± 1.05	− 0.03 ± 0.05	1	− 0.03
	I-P	− 1.42 ± 1.11	− 1.43 ± 1.06	0.01 ± 0.04	1	0.01
CA (J0)	P-S	0.53 ± 0.62	0.49 ± 0.63	0.04 ± 0.03	0.75	0.06
	I-S	0.52 ± 0.63	0.49 ± 0.63	0.03 ± 0.03	1	0.04
	I-P	0.52 ± 0.63	0.53 ± 0.62	− 0.01 ± 0.03	1	− 0.02
CA (45)	P-S	− 0.05 ± 0.36	− 0.03 ± 0.35	− 0.02 ± 0.02	0.81	− 0.05
	I-S	0.01 ± 0.39	− 0.03 ± 0.35	0.04 ± 0.02	0.06	0.11
	I-P	0.01 ± 0.39	− 0.05 ± 0.36	0.06 ± 0.02	< 0.001*	0.16

^a^Each measurement; ACD: Anterior Chamber Depth, WTW: White-to-White diameter of cornea, CCT: Central Corneal Thickness, Sim-K1: Simulated Flat Keratometry, Sim-K2: Simulated Steep Keratometry, Sim-Km: Simulated mean Keratometry, CA: Corneal Astigmatism, CA (J0): Regular component of Corneal Astigmatism (0°/90°), CA (45): Oblique component of Corneal Astigmatism (45°/135°); all keratometry values are reported as diopter, ACD, and WTW values are reported as mm, and CTT values are reported as micrometer.

^b^P: Pentacam HR, S: Sirius, I: IOLMaster 700.

^c^Mean ± SD (standard deviation) measured by each device.

^d^Mean Difference ± Standard Error.

^e^Bonferroni corrected *P* values; asterisk (*) indicates a significant *P* value (< 0.05). (The repeated-measures ANOVA tests were initially conducted for all of the measurements, obtaining a significant *P* value < 0.001. As a result, the pairwise comparisons were subsequently performed using the paired-samples t-test).

^f^Effect size.

## Discussion

The agreement and possible interchangeability of measurements derived from various imaging systems are of great importance for ensuring the validity of treatment planning and avoiding repeated scans. However, limited evidence exists on the possibility of arithmetic adjustments in the case of poor inter-device agreement. Our results showed that, from a clinical perspective, Pentacam HR, Sirius, and IOLMaster 700 measurements of CCT and ACD may be used interchangeably, particularly for IOL power calculations. However, WTW showed greater variability, with poor to moderate consistency across devices. However, applying an arithmetic adjustment for WTW between Pentacam HR and Sirius yielded excellent agreement, unlike IOLMaster 700–Pentacam HR and IOLMaster 700–Sirius. This could be a leap forward in approaching interchangeability between WTW measurements; however, true clinical interchangeability remains ahead and should also be reflected in the LoA. Caution is warranted for deciphering the interchangeability of keratometry measurements. Notwithstanding their excellent consistency across pairwise device comparisons, the pairwise LoA are relatively large and may thus render individual measurements non-interchangeable.

This great variability in unadjusted WTW measurements has been well-documented in the literature^26^. Horizontal WTW was traditionally used as the main estimator of horizontal sulcus-to-sulcus (STS) distance, which is the most critical parameter in phakic IOL size estimation. Although internal distances, like STS and angle-to-angle (ATA) distances, were always preferred over WTW for this purpose, WTW is still widely used owing to its widely available reports and its employment, along with internal ACD, in STAAR Surgical Online Calculation and Ordering System (OCOS™, Staar Surgical, USA) to predict the best lens size^13,27^. Most complications following ICL surgery occur due to inappropriate sizing of the ICL. Oversizing may lead to inadequate vaulting and hence pupillary block, angle closure, elevated intraocular pressure (IOP), and malignant glaucoma, whereas implantation of an undersized lens is a risk factor for developing anterior subcapsular cataract or zonular damage combined with ICL dislocation^28,29^. The estimation of ICL size usually involves adding a constant value, ranging from 0.5 to 1 mm, to WTW^30^. Nonetheless, previous studies raised concern regarding the inter-device variability of WTW measurements and the weaker correlation between WTW and STS among particular populations^26,31^. A recent study by Huang et al. demonstrated that the correlation between WTW and STS becomes less reliable in patients with an AL > 28.5 mm, suggesting that the anterior chamber width (ACW) is a better predictor than WTW or ATA^31^. Furthermore, there is growing evidence that automated systems provide a larger WTW compared to surgical calipers^13,27^. Still, there are discrepancies in WTW measurements across various imaging systems, utilizing a wide range of different technologies^27^. For instance, Salouti and colleagues reported WTW measurements of 11.72 ± 0.45 mm and 11.41 ± 0.42 mm for the IOLMaster 700 and Pentacam HR, respectively (*P* value < 0.001; LoA: [− 0.17: 0.78])^32^. Ferrer-Blasco and associates reported that IOLMaster 700 recorded significantly higher WTW measurements compared to Sirius (12.18 ± 0.40 vs. 11.90 ± 0.37 mm; LoA: [− 0.10: 0.65])^33^. Abdi and associates demonstrated that Sirius presented significantly higher WTW values than Pentacam (12.26 ± 0.39 vs. 12.03 ± 0.41), revealing an MD of 0.23 ± 0.12 (ICC = 0.95; LoA: [− 0.47: 0.01])^34^. A recent systematic review by Muzyka-Woźniak and associates investigating the agreement of WTW measurements across 41 studies involving 19 ocular biometers and 4595 eyes also revealed that the MD in WTW measurements between devices differed from 0.01 mm to 0.96 mm, and the LoA widths varied between 0.31 and 2.45 mm (median: 0.65 mm), requiring significant consideration when planning for phakic IOL surgeries^26^. These discrepancies in WTW measurement across automated systems may arise from the inherent difficulty in precisely identifying the cornea–sclera boundary^27^; however, we hypothesized that these disparities may also follow a fixed systematic bias that could be adjusted through adding a constant, as was shown between Pentacam HR and Sirius, according to our findings. Interestingly, a similar practice was traditionally used to predict ICL size based on WTW (typically 0.5–1 mm) and could partly address inter-device agreement and internal distance correlation issues. Nevertheless, larger studies across different systems and populations are needed to confirm this observation.

Other than WTW, our findings indicated nearly excellent inter-device agreement for CCT and ACD. Song and associates recently conducted a study on the consistency of CCT measurement in 269 healthy myopic patients using the same devices as the current study and found that the mean CCT measurements were reported as 541.63 ± 31.67 μm, 541.74 ± 33.36 μm, and 548.90 ± 34.19 μm by Pentacam, Sirius, and IOLMaster 700, respectively^35^. Unlike the Pentacam-Sirius pairwise comparison, significant differences were detected when comparing IOLMaster 700-Sirius and IOLMaster 700-Pentacam. However, they also reported that the measurements were highly correlated, with Pearson’s r exceeding 0.96 across all pairwise comparisons. Their findings align with ours, as IOLMaster 700 produced slightly higher CCT values compared with the other devices. Excellent pairwise correlations also existed between the measurements. However, it’s imperative to highlight that there are some considerations when interpreting the statistical findings. The absolute agreement was evaluated in their study using pairwise comparisons of mean measurements, resulting in significant differences between IOLMaster 700 measurements and those from other devices. As a principle, statistical tests tend to detect even minor differences as significant as the sample size grows larger. Therefore, it is recommended to provide the effect size to give a better understanding of practical rather than statistical significance.

Our results regarding the agreement of ACD values align with the current scholarly literature. Another study by Song and colleagues recently reported mean ACD measurements with the Pentacam, Sirius, and IOLMaster 700 systems as 3.26 ± 0.26 mm, 3.30 ± 0.26 mm, and 3.22 ± 0.25 mm, respectively^36^. Our findings also showed that Sirius and IOLMaster 700 had the greatest and lowest mean ACD measurements, respectively (3.41 ± 0.36 mm and 3.36 ± 0.36 mm). Regarding their clinical interchangeability, a review by Domínguez-Vicent and colleagues confirmed that agreement studies on a large number of imaging system combinations, including Pentacam-IOLMaster 700, showed clinically acceptable LoA, particularly for IOL power calculation^37^. However, one should bear in mind that, as Bland and Altman stated, methods that demonstrate sufficient agreement for one application may not exhibit the same level of agreement in another^38^.

It is suggested that differences of less than 0.25 D in refractive outcomes could be considered as clinically acceptable in agreement studies^39,40^. Notably, according to the Kane IOL power formula, a change of 0.18 mm in ACD and 30 μm in CCT also translates to 0.10 D and 0.04 D in refractive power, respectively. Interestingly, in their review of 66 studies, only three reported an inter-device MD between ocular biometric parameters, corresponding to a difference greater than 0.25 D in the implanted IOL^41^. The LoA for CCT and ACD was a maximum of ± 25 μm and ± 0.2 mm across any pairwise devices, respectively. This conclusion is also supported by high ICC values exceeding 0.95 and a relatively small Hedges’ g effect size. These values correspond to clinically acceptable variations according to the mentioned formula and may thus be considered interchangeable for that task. The current literature supports our findings, as these parameters were repeatedly shown to be highly correlated between systems that are based on the Scheimpflug camera and SS-OCT technologies, with varying and mostly clinically minor discrepancies in their mean measurements^35,36,39,42–46^. Nevertheless, the statistical findings should never replace the clinical or case-by-case considerations.

Our results regarding keratometry values show more variability. Correspondingly, comparing Pentacam AXL and IOLMaster 700, Saadettin and associates reported a significant increase of 0.23 ± 0.15 D in mean keratometry (LoA: [− 0.52: 0.07]) and 0.04 ± 0.02 mm in ACD measurements (LoA: [0.00: 0.09]) when using Pentacam AXL, suggesting that, contrary to the ACD, mean keratometry cannot be used interchangeably. Notably, the CA (J0) and CA (J45) were not significantly different^47^. Moreover, Shajari and associates showed no significant differences between the flat and steep radii of the anterior surface of the cornea (FRf and FRs, respectively) between the Pentacam AXL and IOLMaster 700^48^. Another study by Abdi and associates compared measurements derived from Pentacam HR and Sirius, revealing a good to excellent agreement in the ACD (ICC = 0.82; LoA: [− 0.38: 0.40]), FRf (ICC = 0.98; LoA: [− 0.02: 0.18]), FRs (ICC = 0.97; LoA: [− 0.05: 0.15]), CA (ICC = 0.95; LoA: [− 0.66: 0.28]), and mean keratometry (ICC = 0.98; LoA: [− 0.24: 0.70]) measurements^34^. Similarly, Lu and associates evaluated the repeatability and agreement of anterior segment measurements, including flat, steep, and mean keratometry, as well as CA and Jackson cross-cylinder vectors, ACD, CCT, aqueous depth, and WTW between Sirius and IOLMaster 700. They concluded that there was high intraobserver repeatability and inter-device agreement, and that their measurements could be used interchangeably. However, they noted that in spite of narrow LoA widths, IOLMaster 700 reported keratometry and corneal diameter measurements that were 0.1 D and 0.1 mm higher than Sirius, respectively^49^. In another study, Lee and colleagues assessed inter-rater agreement between an SS-OCT tomographer (Casia SS-1000) and a combined dual Scheimpflug-Placido disc-based tomographer (Galilei G2) between two groups of normal and post-refractive myopic patients. The two systems utilize relatively the same technology as IOLMaster 700 and Sirius. Interestingly, their findings revealed that these two systems showed excellent agreement in terms of anterior flat, steep, mean keratometry, and CCT measurements in both groups (ICC > 0.98 for all), which was in line with our findings^50^.

Another study by ÖZYOL et al. on the agreement of Pentacam HR and IOLMaster 700 measurements revealed that although no significant difference was observed for the CA (J0), CA (J45), CCT, and ACD measurements, IOLMaster 700 produced significantly higher average keratometries than Pentacam HR (43.2 ± 1.3 vs. 43.0 ± 1.3) with a LoA ranging from − 0.38D to − 0.02D, suggesting that these measurements are not interchangeable^51^. Similarly, our findings support that IOLMaster 700 significantly overestimated the keratometry parameters compared with Pentacam HR, and the LoA were − 0.64 to 1.01D, − 0.40 to 0.78D, and − 0.32 to 0.72D for Sim-K1, Sim-K2, and Sim-Km, respectively. The judgment on the interchangeable use of these keratometry measurements is nuanced. Although the ICC(2,1) and ICC(3,1) values exceeded 0.95 for all keratometry measurements, we found LoAs of − 0.80 to 0.43 D and − 0.32 to 0.72 D when comparing Sim-Km values between Pentacam HR-Sirius and IOLMaster 700-Pentacam HR, respectively. With considerable improvements in cataract surgeries over the decades, prediction error (PE) following IOL implantation surgery mostly falls within the ± 0.25 D and ± 0.50 D thresholds^52–55^. This makes such differences in Sim-Km values noteworthy and clinically not interchangeable.

Besides precise biometry and improved IOL power formulas, constant optimization has also been introduced to further reduce median absolute error following IOL implantation surgeries^56^. Constant optimization refers to the process aimed at achieving a mean PE of zero in an analyzed sample of patients when evaluating the accuracy of IOL power formulas. This step is essential for eliminating any systematic biases originating from factors such as the physical properties and design of the IOL, biometric devices, and the surgical techniques^16,57^. Similar to how IOL factors affect the accuracy of IOL power formulas, slight variations in readings across different systems could be attributed to differences in imaging techniques, processing algorithms, and device calibration status, all of which may follow systematic trends and can even heavily affect inter-device agreement. Furthermore, accommodation effects from varying light sources might impact the accuracy of some of the parameters^58^. We tried to first evaluate whether the observed differences reflect random variability or systematic bias using two types of ICCs and check to what extent a poor agreement could be adjusted using the MD, a practice inherently similar to constant optimization. These findings underscore the importance of comprehensive systematic evaluation when interpreting agreement across imaging systems and establish a practical framework for future studies to more accurately assess agreement between readings derived from imaging systems.

To the best of our knowledge, this is the first study to assess agreement and consistency of measurements across three established imaging systems using effect size calculations, as well as Bland-Altman and dual ICC analyses. The potential for adjustment between instruments was further explored by examining the disparity between ICC(2,1) and ICC(3,1). However, our study had several limitations. Firstly, it was limited to otherwise healthy ametropic eyes. Therefore, evaluating the agreement of measurements in pathological conditions, such as corneal ectatic disorders or post-refractive surgery, would be valuable. Moreover, although the disparity between the ICC(2,1) and ICC(3,1) rises from a fixed systematic bias equal to the mean difference of measurements, their arithmetic adjustment could be more complex and might depend on the magnitude of measurements, necessitating the need for special regression analyses, which lie beyond the scope of the current study. A greater sample size could also enhance the reliability of agreement analysis and more precisely determine the mean difference, which plays a central role in arithmetic adjustments. Therefore, it is recommended that future agreement studies cover a wide range of imaging systems, examine inter-device variability to determine whether it is randomly distributed or follows a systematic pattern, explore optimal strategies for arithmetic adjustment, and consider clinical implications rather than pure statistical significance when interpreting the findings.

## Conclusions

In conclusion, the ACD and CCT may be used interchangeably across these three systems for tasks like IOL power calculation. On the other hand, the keratometry parameters may not be used interchangeably due to their wide pairwise LoA, especially between Pentacam HR-Sirius and IOLMaster 700-Pentacam HR. Despite the wide LoA, the agreement of WTW measurements between Pentacam HR-Sirius could be remarkably increased through an arithmetic adjustment. In contrast, this adjustment was not feasible between the IOLMaster 700 and either Pentacam HR or Sirius. Clinicians should take these discrepancies into account, be aware of device-specific measurement biases, and exercise caution when combining or comparing biometric data across various imaging platforms.

## Data Availability

The data supporting the results of this study are not publicly available due to privacy concerns regarding the participants. However, they can be accessed from the corresponding author upon reasonable request.
